# Creating Age-Friendly Health Systems: Age Matters

**DOI:** 10.1093/geroni/igaa057.3193

**Published:** 2020-12-16

**Authors:** Terry Fulmer

**Affiliations:** The John A. Hartford Foundation, New York, New York, United States

## Abstract

Since 2015, The John A. Hartford Foundation has been funding strategies to create Age-Friendly Health Systems (AFHS). Led by the Institute of Healthcare Improvement, in partnership with the American Hospital Association and the Catholic Health Association, the AFHS movement is rapidly growing, with participation in all 50 states from over 450 sites, including the full continuum of care settings. Partnerships with private and public entities are accelerating the work. As one example, the Health Resources and Services Administration has embedded AFHS principles into the Geriatrics Workforce Enhancement Program. This Kent Lecture will focus on the genesis and trajectory of the AFHS social movement and discuss how the effort will lead to an age-friendly ecosystem that transcends boundaries and cultures and leads to a common framework for the way we approach care, caregiving and communities for optimizing the lives and wellbeing of all older adults.

